# Psychiatric effects of malaria and anti-malarial drugs: historical and modern perspectives

**DOI:** 10.1186/s12936-016-1391-6

**Published:** 2016-06-22

**Authors:** Remington L. Nevin, Ashley M. Croft

**Affiliations:** Department of Mental Health, Johns Hopkins Bloomberg School of Public Health, 624 N. Broadway, Room 782, Baltimore, MD 21205 USA; School of Pharmacy and Biomedical Science, University of Portsmouth, James Watson Building (West), Portsmouth, Hants PO1 2FR UK

**Keywords:** Malaria, Malariotherapy, Anti-malarial drugs, Psychiatric effects, Toxicity

## Abstract

The modern medical literature implicates malaria, and particularly the potentially fatal form of cerebral malaria, with a risk of neurocognitive impairment. Yet historically, even milder forms of malaria were associated in the literature with a broad range of psychiatric effects, including disorders of personality, mood, memory, attention, thought, and behaviour. In this article, the history of psychiatric effects attributed to malaria and post-malaria syndromes is reviewed, and insights from the historical practice of malariotherapy in contributing to understanding of these effects are considered. This review concludes with a discussion of the potentially confounding role of the adverse effects of anti-malarial drugs, particularly of the quinoline class, in the unique attribution of certain psychiatric effects to malaria, and of the need for a critical reevaluation of the literature in light of emerging evidence of the chronic nature of these adverse drug effects.

## Background

This review, written for the psychiatrist as much as for the malariologist, serves as an introduction to what by modern perspectives may be viewed as surprising areas of common interest. The disciplines of psychiatry and malariology were united briefly in the early to mid twentieth century in the historical practice of malariotherapy, in which patients were intentionally inoculated with malaria for its presumed therapeutic neuropsychiatric effects. Although this practice has long fallen out of favour and although the two disciplines have subsequently diverged, insights from malariotherapy and more broadly the field of malariology have continued to make important contributions to psychiatry in the modern era.

Untreated or delayed treatment of malignant *Plasmodium falciparum* malaria infection may result in coma. This cerebral complication of malaria is implicated particularly with lasting cognitive deficits that owing to the high prevalence of disease worldwide may contribute significantly to the global burden of psychiatric morbidity [1]. Yet similarly, anti-malarial drugs, particularly of the quinine-like class of quinolines commonly used in treatment and prevention of disease, are also increasingly recognized to exert potentially chronic psychiatric adverse effects that may also contribute to this global burden of morbidity [2].

This review, inspired by an earlier seminal work [3], synthesizes over a century of critical literature from psychiatry and malariology—ranging from anecdotal historical observations to evidence from modern randomized controlled trials—to explore the evolving understanding of the psychiatric effects of malaria and of anti-malarial drugs. In this review, the evolution of psychiatric effects attributed to malaria is described, from the historical perspective in which a broad range of symptoms were attributed to the disease, to the current understanding of the more limited psychiatric effects of cerebral malaria (CM) and post-malaria syndromes. Next, malariotherapy and its historical role in psychiatry are described. Lastly, the psychiatric effects of anti-malarial drugs, particularly of the quinoline class, are reviewed, including a discussion regarding the potential confounding effect that these may have had on symptoms historically associated with malaria.

### Psychiatric effects of malaria

Although early descriptions of the psychiatric effects of malaria first appeared in the ancient medical literature of Hippocrates and Galen [4, 5], it was only many centuries later that descriptions of these effects became relatively common. Such descriptions grew particularly detailed in the aftermath of World War I (WWI), where malaria, epidemic on the Macedonian front between 1916 and 1918, was unexpectedly found to be the leading cause of psychiatric disorders among soldiers of the Allied powers fighting there [6].

Detailed reports in the literature from this era described a seemingly bewildering set of psychiatric effects attributed to both severe and mild forms of the disease. Early reports had described dreamlike states [7], anterograde amnesia, difficulties with concentration, disorientation, and forgetfulness [8], confusional psychosis, dementia, “melancholia”, and mania [9] emerging in patients recovering from severe malaria. During this era, mild chronic or intermittent malaria infection had also become associated with symptoms of “neurasthenia”, including depression, irritability, anxiety, and insomnia [10]. In subsequent years, even more severe symptoms such as confusion [11], intermittent psychoses, and delirium were also commonly attributed to those with mild forms of malaria [12].

This literature even warned of the potential for missed diagnoses of malaria prior to the onset of malarial fever among psychiatric patients admitted for care and thought to be suffering from delirium and psychoses [13]. Many such patients were diagnosed with malaria only some time later at the onset of fever, or when their symptoms progressed to coma. These reports described a common prodrome of hallucinations, anxiety, crying, violence, agitation, and a dreamy and confusional state [13].

So common and diverse became the psychiatric effects attributed to both severe and mild forms of malaria during the post-WWI era, that an entire textbook, well received by psychiatrists at the time [14], was published with chapters featuring case reports of malarial psychoses, “dementia praecox”, confusional and delusional insanity, mania, “melancholia”, delirium, and dementia attributable to the disease. As noted by the author in his preface, “very few known syndromes are absent from the list apparently arising from malarial infection” [15].

By World War II (WWII), growing US and allied military experience, particularly in the South Pacific, had identified similar psychiatric effects associated with malaria. Reports from US military clinicians noted a characteristically confusional form of psychosis, as well as persecutory, grandiose, bizarre delusions, and symptoms of anxiety, depression, and mania associated with both severe and mild forms of the disease [16, 17]. Yet others had begun to challenge the true association of many of these symptoms with malaria, noting “too great a tendency to blame malaria for many of these symptoms” [16], with the temporal association of certain of these symptoms suggesting to other authors a toxic process [16].

Despite such growing suspicions, the psychiatric effects of malaria were still considered “more common than are generally known” [4], and a military journal editorial noted that malaria should be considered “associated with protean mental states” [18]. Psychiatric symptoms also remained clearly associated in the literature of the era with even the mild chronic and relapsing forms of disease, with a case report describing psychosis developing weeks prior to the onset of fevers from the generally benign *Plasmodium vivax* malaria [17].

Reports from among soldiers of the Vietnam War era similarly described mostly transient cases of psychosis, and impaired memory and concentration generally following admission for treatment for malaria [19], often lagging admission by some days, and typically without evidence of such symptoms while previously febrile [20]. Neuropsychological testing of certain of these patients both soon after their acute illness and following their recovery identified common patterns of deficits, particularly in tests of recent memory, visual organization, psychomotor speed, and visual motor integration [21, 22]. Decades later, follow-up of malaria patients from the Vietnam War demonstrated lasting changes in personality, depression, and symptoms of partial seizure [23], as well as deficits on tests of memory, and other evidence of chronic cognitive dysfunction on neuropsychological testing [24].

In more recent studies of broadly defined malaria infections, acute malaria among children [25], and multiple recurrent malaria infections have been associated with adverse effects on short-term cognitive performance [26]. Similarly, in recent reviews, among chronic psychiatric effects, the most common and strongest evidence exists for a risk of chronic cognitive impairment following successful malaria treatment [27–30]. Additional retrospective studies have attributed a risk of subclinical depressive and anxiety symptoms [31], as well as altered executive functioning [32], particularly following severe malaria infection.

### Cerebral malaria

Unlike past usage of the term, which had been significantly broader in scope [33], and had included presentations that had involved organic mental syndrome and even acute personality change [21], the modern strict definition of CM has gradually evolved to require alterations in consciousness for diagnosis [34, 35]. This modern definition of CM, which was developed for research purposes [36], and which is considered a diagnosis of exclusion [37], specifically limits use of the term to a patient with coma occurring in the presence of malignant *P. falciparum* malaria parasitaemia [38]. The strong and highly sensitive and specific association of such clinically-defined CM with objective signs, including malaria retinopathy, has significantly improved diagnostic reliability [39, 40], particularly among children in tropical countries who are primarily affected by the disorder.

Unlike historical definitions, the modern definition of CM is primarily described as an acute neurological disorder and is notably less associated in the literature with acute psychiatric symptoms [33, 41]. Some authors have questioned its acute association with psychosis [42], although early case reports [43] and reviews [44] through the 1990s continued to describe self-limiting acute psychosis occurring following treatment for CM.

Prospective follow-up of patients specifically with CM are limited, but small studies of children find increased risk of cognitive deficit [45] lasting through 2 years of follow-up [46]. In children surviving CM confirmed by retinopathy, certain measures of retinopathy were associated with chronic deficits in attention and cognitive ability [40]. A prospective study of children with a spectrum of severe malaria including CM also found sub-acute behavioural problems and decreased attention [47].

Retrospective studies of children with CM also report long-term impairments in cognition and executive functioning [48] as well as developmental and cognitive impairments including in speech, language, and memory [49–51], with seizure during acute infection emerging as a strong risk factor [52]. Small retrospective studies in adults have also shown chronic impairments in verbal learning and abstract reasoning [53].

With the rise in diagnostic specificity of malaria disorders, investigators have been able to distinguish chronic sequelae of treated CM from those of other complications of malaria, including severe malarial anaemia (SMA) without coma. Consistent with the findings in other anemic disorders, children under 5 years of age with SMA have also been found to suffer long-term cognitive impairment [54].

### Post-malaria syndromes

With the evolution of the definition of CM requiring neurological signs during acute *P. falciparum* parasitaemia for diagnosis, three additional disorders have been defined to describe cases in which neuropsychiatric sequelae become manifest primarily following treatment and resolution of acute malaria infection, independent of the severity of prior disease, including occasionally occurring following illness from benign *P. vivax* infection. One disorder has been referred to as delayed cerebellar ataxia (DCA) [55], or in some cases as related delayed cerebellar syndromes [56, 57]. A later somewhat distinct disorder has been referred to as post-malaria neurological syndrome (PMNS) [58] while a related disorder of acute disseminated encephalomyelitis (ADEM) has been defined which is diagnosed with the aid of neuroimaging studies. Although disagreement has been raised as to whether certain of these constitute unique disorders [59], they appear to differ from each other and from the modern disorder of CM in regards to the latency, frequency, and type of reported psychiatric findings.

Although psychiatric symptoms are only rarely associated with delayed cerebellar syndromes in the literature, when reported, these have included giddiness [56], alterations in attention [60], and word-finding difficulties [61].

Similarly, in cases of ADEM, although neurological findings tend to predominate, psychiatric symptoms including alterations in sensorium [62], confusion [63], disorientation [64], and language difficulties have been reported [65].

In contrast to ADEM and the delayed cerebellar syndromes, psychiatric symptoms have been far more commonly reported with PMNS, to include the broader range of symptoms historically associated with malaria infections, including anxiety [66], temporospatial disorientation [67], difficulties with word-finding [68], acute confusion [69], mood changes, [70], psychosis with hallucinations, persecutory delusions, and mania [58], and disturbances in behaviour [71].

### Malariotherapy of psychiatric disease

Ancient knowledge of the psychiatric effects of malaria included accounts describing reductions in aggressiveness among the mentally ill after bouts of malaria [72]. In the early twentieth century, growing awareness of the reported psychiatric effects of malaria, and recognition that febrile illness had proven therapeutic in the treatment of neurosyphillis [73]—a debilitating and epidemic condition then known as “general paresis” for which no effective treatments were then available [74]—prompted the first formal experimentation with the practice of malariotherapy.

According to the first published description of the practice [75], for which its author would subsequently win the Nobel Prize in medicine [76–78], neurosyphillis patients were intentionally inoculated with blood-stage benign forms of malaria, allowing upwards of ten cycles, or paroxysms, of intermittent fever prior to treatment of the infection with available anti-malarial drugs [75].

Early case reports of this technique described common and pronounced improvements in psychiatric symptoms including reduced irritability, aggression, mania, psychosis, confusion, amnesia, and disorientation following such fever treatment [75, 79–84]. Complete cures, later categorized as remissions by some authors [85, 86], were initially reported in up to a third of patients. Partial cures were reported in about a half [87], although subsequent reports claimed somewhat greater risks and more mixed success rates [86, 88–93]. Intriguingly, following the termination of fever paroxysms by treatment with available anti-malarial drugs, a distinct mental state described as “paraphrenic” or late psychosis was occasionally described with transient confusional, delusional, incoherent, hallucinatory, and manic-depressive features [94].

So common became the practice of malariotherapy soon after its introduction that concerns of autochthonous spread of malaria led to health ministries establishing standard guidelines for the practice [95], including the enforcement of vector controls in the vicinity of patients [96, 97]. Although mosquito inoculation, rather than blood-stage inoculation, had been recognized as associated with a risk of malaria relapse [98], and to thus pose an increased risk of autochthonous spread, the growing popularity of malariotherapy for the treatment of non-syphilitic psychiatric patients [99], together with concerns regarding cross-contamination with *Treponema pallidum*, led to increasing use of direct mosquito inoculation for the purpose [85, 100, 101].

Subsequent experience would demonstrate that the results of treatment of other psychiatric disorders (including chronic “dementia praecox” or schizophrenia, as well as manic-depressive psychosis) were mixed and perceived as less successful [73, 94] and with the development of effective antibiotics for treatment of neurosyphillis, the practice of malariotherapy gradually fell into disuse.

### Psychiatric effects of anti-malarial drugs

Although many classes of drug have wide clinical applications as anti-malarials and also are known to cause psychiatric effects, the quinoline derivatives are unique in medical history for having been used ubiquitously to treat and prevent malaria, often to the exclusion of other classes of drug. For example, with few alternatives, prior to WWI, malaria was invariably treated and prevented with quinine, or occasionally with one of the other naturally occurring quinoline-based cinchona alkaloids [102, 103]. Similarly, in the aftermath of WWI and through WWII, the only other anti-malarials that came into reliable use were synthetic quinolines initially developed by German scientists [102].

It was only in the decades following WWII, with the gradual development and deployment of drugs of alternative classes, to include proguanil and pyrimethamine [102], the discovery of the anti-malarial activity of tetracyclines [104, 105], and recently, the isolation of naturally-occurring *qinghaosu* [106], or artemisinin, from *Artemisia annua* [107, 108], that effective and widely used alternatives to the quinolines emerged both for prevention and treatment of disease.

While the quinolines have remained in common use through the present day, unlike these newer anti-malarial classes, the utility of certain members of the quinoline class in treating and preventing malaria has become progressively compromised through rising awareness of symptoms of idiosyncratic intoxication and central nervous system (CNS) toxicity associated with their use. Suggestive of a broad class effect spanning common structural variations, anti-malarial quinolines with psychiatric effects that are commonly described in the literature include the naturally occurring 4-quinolinemethanol cinchona alkaloids quinine and quinidine, whose use as anti-malarials predominated prior to WWII; the synthetic acridine derivative quinacrine (which may be considered a ring-substituted 4-aminoquinoline), whose use reached a peak during that war; the synthetic 4-aminoquinolines including chloroquine, whose use expanded widely following WWII; the synthetic 4-quinolinemethanol mefloquine, developed in the aftermath of the Vietnam War and whose use took the place of chloroquine throughout the 1980s and 1990s, and the synthetic 8-aminoquinolines, which have been used sporadically since their first development following WWI, but which are now being considered for expanded use in global malaria control efforts.

### Quinine and the cinchona alkaloids

The earliest human experience with the quinolines dates to discovery of the naturally-occurring drug quinine, a 4-quinolinemethanol alkaloid first isolated from the bark of the *Cinchona* tree in the seventeenth century, together with the related cinchona alkaloid quinidine [109]. Although reports from the early twentieth century noted that quinine could have significant psychiatric effects [11], including causing depression, mania, irritability and personality change [110], and that these might not be distinguished from those attributed to disease [7], only relatively recently has it become clear that quinine, independent of malaria infection, may cause a serious toxic state marked by symptoms including delirium and confusion [111, 112].

In contrast, a broader range of psychiatric effects specifically due to quinidine have been widely reported in the literature, including mood change [113], auditory hallucinations [114, 115], visual hallucinations and delusions [115, 116], paranoia [116, 117], confusion, and delirium [116, 118].

### Quinacrine

Quinacrine, a derivative of acridine dye first synthesized in the early 1930s [119], marketed initially under the names erion and atabrine [120], then later also under the name Mepacrine [121], may be considered a ring substituted 4-aminoquinoline. In early reports of its use, it was noted to induce “definite cases of delirium and mental disturbances” [122], including symptoms of depression [123], and delusional insanity. Case reports described acute personality and behavioural change, severe insomnia and anxiety described as an “impression of impending death” [124], visual hallucinations, and cognitive dysfunction. One patient complained that “a large part of his brain was out of action” [124], while another patient was described as manic, restless, disoriented, and suffering visual hallucinations that “snakes and fantastic animals were crawling on the floor” [125].

Although early marketing materials for the drug acknowledged “mental disturbances which have been observed in a few instances during treatment” [126], it was also emphasized that disorders of the nervous system, including sudden delirium and delusional insanity, were “not infrequent in the course of acute malarial fevers” [126], and that it was “important to distinguish between those due to malaria itself and those which may be attributable to the drug” [126].

With shortages of quinine during WWII, quinacrine was added to the US pharmacopoeia in 1942 [127], and was quickly prioritized for military use both as treatment and as a suppressive prophylaxis throughout the war [128]. Although studies of the acute toxicity of quinacrine had been planned during a wartime anti-malarial drug development effort [129], these were soon deprioritized after they were declared to be of “minor importance” to military leadership [130]. Although early studies in the UK had suggested that quinacrine caused “troublesome dreams” [131], and independent studies in the US had noted slight increases in nervousness [132], it was initially unclear whether such presumed toxic effects were actually due to quinacrine or merely to a contaminant [128, 133].

Towards the end of the war, however, sufficient experience had accumulated, including from rechallenge experiments, for wartime researchers to conclude that quinacrine use was definitely associated with a toxic psychosis distinct from the effects of malaria, often of abrupt onset [134, 135], described as including “mania, catatonia, and paranoid and hallucinatory states” [136], and typically developing only several days after the start of treatment [134]. Authors noted the psychosis was typically marked either by “excitement, hallucinations, and delusions” with an affect of “euphoria and expansiveness” [137], or alternatively with “retardation, disorientation, and amnesia to recent events, together with confabulation” [137], where affect was predominantly “one of bewilderment and fearfulness” [137].

In controlled tests of intellectual functioning, a large proportion of subjects administered quinacrine were found to suffer significant subclinical impairment, and in two cases of psychosis that developed subsequently, tests of intellectual functioning had showed mild impairment prior to onset [138]. The authors concluded that “among a large group” administered quinacrine, “some would show clinical confusion” [139]. A separate controlled study conducted among healthy US military physicians clearly demonstrated quinacrine induced changes in the electroencephalograph [140], accompanied by apprehension, restlessness, insomnia, “awakening dreams which at times were of frightening and nightmare quality” [141], and symptoms of mania described as “an unusual push of activity” associated with “tension, irritability, and anxiety” [142]. One subject experiencing panic attack and flight of ideas noted he “felt so bombarded by stimuli that he could not deal with them effectively” [141]. The patient became very anxious, depressed, confused, and was thought to be suffering delirium, although his consciousness was in fact preserved [141, 142].

With the publication of such research findings initially restricted due to wartime considerations, official documents acknowledged only that “psychological disturbances occasionally appear” with use of quinacrine [143], and that the drug could cause “mild excitement” and rarely, “acute maniacal psychoses” [144]. Despite the imprecision of official reports, quinacrine was nonetheless recognized by numerous wartime physicians as a potential cause of psychiatric disorders [145], and as the war ended and in subsequent years as restrictions were rescinded, numerous publications appeared describing psychiatric effects associated with the drug’s use.

One ecological study linked an increase in a combat unit’s rate of psychoses to their initiation of quinacrine for prophylaxis [146]. A case report described a previously healthy soldier suffering three distinct episodes of mania, each occurring with recurrent use of quinacrine both for prophylaxis and treatment [147]. Another detailed case report described a patient initially free of psychiatric history and thought to be suffering from cerebral malaria, who following 5 days of treatment with quinacrine developed excitement, talkativeness, and rambling, progressing to depression, impaired concentration, disorientation in time and place, anterograde and retrograde amnesia, and delusions. These symptoms lasted 2 weeks [148]. One case series of those treated with quinacrine reported psychoses developing 0–10 days after the last day of malarial fever [149]; another between 3–12 days following the start of quinacrine [137]; while another reported an even wider latency of onset of 2–22 days, with a similarly wide range in time to satisfactory recovery of 1–30 days. Of significance, in at least four cases in the series, failure to recover and the development of chronic psychiatric disorders was noted [135].

In the immediate postwar years, a few additional studies and case reports of psychiatric effects from quinacrine were also published [150], including a series describing inappropriate sexual behaviour associated with psychoses [151]. In a final published study of 31 previously healthy Army Medical Department officers who volunteered to take the drug experimentally at treatment doses [152], 77 % suffered some form of mental disturbances: 71 % complained of abnormal, anxious and disturbing and rarely terrifying dreams; 58 % developed sleep disturbances, with loss of up to half of normal sleep, prompting sedative use to treat insomnia in two-fifths of these, or 26 % of the total; 39 % went on to develop severe psychic disturbances, of which a quarter, of 10 % of the total, suffered frank psychoses with delusions, hallucinations, and profound changes in mood, including mania; 35 % complained of tension; 32 % complained of restlessness, and 29 % depression. During an earlier phase of the trial when the drug had been administered at lower prophylactic dose rates, 29 % had noted mild complaints, most significantly abnormal dreams and sleep disturbances, which were deemed “the most reliable warning sign” of the development of “more severe reactions” [152]. Intriguingly, it was reported that during earlier widespread use of the drug, such “minor toxic symptoms of restlessness, insomnia, and increased dreaming might frequently have passed unnoticed as a manifestation of malaria” [152].

### Chloroquine and other 4-aminoquinolines

Chloroquine, a 4-aminoquinoline, was also first synthesized in the 1930s by German scientists, who named it resochin [153, 154]. Expecting a quinacrine-like effect in initial clinical testing, presumably owing to the drugs’ structural similarities, its toxicity was described as being “so great in comparison to its effect” [155], that the drug was deemed “too toxic for practical use in humans” [153] and further German development was abandoned. The drug was rediscovered only during the subsequent US WWII drug development effort [154], where it was initially referred to as SN-7618. Chloroquine was investigated during this WWII era effort only after the potential utility of a related 4-aminoquinoline, sontochin, later referred to as SN-6911, was noted following the Allied invasion of Tunisia, where it was found that the drug was undergoing clinical testing by Axis-affiliated French researchers [153].

In the WWII era of US testing, sontochin was noted to induce acute confusional psychosis and nervousness at high doses [156], and in postwar testing, was found to induce a wider variety of psychiatric symptoms, including anxiety, nervousness, a sensation of “being on edge”, irascibility, and irritability at lower prophylactic dose rates [157]. In contrast, during similar testing, chloroquine appeared to have only “minor toxic manifestations” [158], and in published WWII era trials, appeared devoid of psychiatric effects. Considered more effective and less toxic than sontochin during the WWII development effort [153], chloroquine was also considered “better” than quinacrine, and “much better” than quinine [159], and by the early 1950s it had begun to replace both drugs for the treatment and prophylaxis of malaria [160–162].

With increasing use however, chloroquine came to be associated with similar idiosyncratic cases of toxicity as those noted previously with the cinchona alkaloids and with quinacrine and sontochin. These took the form of various psychiatric symptoms including sleep disturbances and insomnia [163–165], mood and personality change [165, 166], impulsivity, flight of ideas, and inappropriate behaviour [165, 167], persecutorial and paranoid delusions [165, 168], delusional misidentification [169, 170], depersonalization [166], visual and auditory hallucinations [165, 171], and mania [172, 173]. Suicidality [167] and completed suicide [174–176] were also not uncommonly reported. Although psychiatric symptoms from chloroquine have been noted to often resemble a brief psychotic or delusional disorder [177–179], on careful evaluation they are more likely to feature visual hallucinations, derealization, restlessness, agitation, and anxiety than other cases of psychosis [180].

A case of severe psychosis with features of depersonalization and anxiety that developed under controlled research settings following chloroquine therapy for induced malaria was causally attributed by the researchers to the drug and not to malaria disease; following resolution of psychosis the patient experienced difficulties with concentration that lasted 4 months [181]. A separate case of transient amnesia linked to the drug, [182], and difficulties with concentration [183], confusion [184], delirium, and catatonia [165, 185] have also been reported.

### Mefloquine

Mefloquine is a synthetic 4-quinolinemethanol analogue of quinine that was developed by the US military amid concerns of rising chloroquine resistance during the Vietnam War [186] through a subsequent drug development effort similar to that of the earlier WWII era [187] that spanned the 1960s–1980s [188]. Although once commonly considered the drug of choice for treatment and prophylaxis of malaria and marketed under the trade name Lariam, use of mefloquine has been severely limited by awareness of significant idiosyncratic CNS toxicity [189] which, as with quinacrine [152], has been found to affect a sizeable minority of users at prophylactic dosages and a majority of users at treatment dosages [189]. According to recent European product labelling [190], very commonly reported psychiatric effects occurring in >10 % of prophylactic users of mefloquine include abnormal dreams and insomnia, while commonly reported psychiatric effects occurring in 1–10 % of prophylactic users include anxiety and depression.

As with earlier generations of quinoline anti-malarials, and consistent with product label warnings of encephalopathy, case reports of psychiatric effects from mefloquine typically describe a complex and diverse range of effects on mood, personality, thought, cognition, sleep, and behaviour [191]. These include asthenia [192], melancholia [193], anxiety [194], phobias [195, 196], feelings of unrest [197, 198], paranoia [199], increased self-esteem [200, 201], impaired judgment [198], social disinhibition [192, 202], giddiness [203], altered sexual libido [204], manic behaviour and paranoid delusions or psychosis [194, 200, 202, 204], hyperreligiosity [204], depersonalization [192, 198, 205], concentration and cognitive problems [197], problems with word finding [201], disorientation [193, 198, 206], symptoms of amnesia and confusion or disorientation [192, 193, 203], sleep disorders [207, 208], severe nightmares, [209] sleep paralysis and “flashbacks” [201], suicidal ideation [210], and suicide [176, 211–213].

On initial presentation, individuals suffering from these diverse effects may appear to be suffering from a potentially wide range of psychiatric disorders that span the diagnostic nosology, to include anxiety, depressive, bipolar, psychotic, dissociative, personality, conversion, and factitious disorders [189, 191, 198]. In particular, the psychiatric effects of mefloquine have been noted to potentially confound the diagnosis of a number of psychiatric disorders prevalent in military settings, including posttraumatic stress disorder [189, 214].

Severe and life-threatening psychiatric symptoms from mefloquine, including suicidal ideation and suicide, may be preceded by often subtle prodromal symptoms including even mild disturbances in sleep and dreaming [215]. Although such symptoms have often previously been attributed to other causes and their critical significance overlooked even by influential authorities [216–218], both the European and US product labelling now explicitly direct the discontinuation of the drug at the onset of any psychiatric symptom [190]. Even the earliest approved US mefloquine product insert appeared to echo the conclusions of prospective studies of quinacrine conducted decades earlier [152] listing “anxiety, depression, restlessness or confusion” as potentially prodromal to a “more serious event” and requiring the drug’s discontinuation [190].

Similarly, while psychiatric symptoms from mefloquine were previously considered reversible and self-limiting, the US product label for mefloquine was recently updated to include a boxed warning (or “black box”) that psychiatric effects from mefloquine could last years after use [219]. A recent follow-up study has found a sizeable proportion of those previously reporting adverse events from the drug complain of long-term psychiatric symptoms including cognitive dysfunction years after use [220]. Mefloquine has been convincingly linked to an increased risk of PMNS [58, 221], and a number of reviews have observed that certain psychiatric symptoms observed following malaria might plausibly be due to this and related quinoline anti-malarials [36, 44].

### 8-Aminoquinolines

The 8-aminoquinolines were among the first synthetic quinoline anti-malarials. Pamaquine (also known as plasmochin), developed by German scientists [222, 223], and in use since the 1920s [224] was initially thought to be free of CNS toxicity, but a fatal case of fatal human toxicity, marked by symptoms of apprehension and restlessness, revealed evidence of extensive brain and brainstem neurotoxicity [225]. By the late 1940s, other synthetic 8-aminoquinoline anti-malarials in common use prior to WWII, or introduced during the WWII drug development effort, had been linked to a severe neurotoxic syndrome [226–228], potentially masking more subtle psychiatric effects, such as previously reported mild psychosis [229, 230].

Primaquine, the only synthetic 8-aminoquinoline to safely emerge from the post-WWII era drug development program, although long considered safe and only rarely associated with psychiatric adverse effects [231], has been linked to rare reports of confusion, amnesia, and depression [232, 233]. Similarly, symptoms reported with the experimental 8-aminoquinoline tafenoquine, originally developed by the US military and now in late-stage development [234] and anticipated for use both as a replacement for mefloquine and primaquine [235, 236] and in mass drug administration campaigns for global malaria control [234] have been described in randomized trials as similar to those of chloroquine [237]. In a recent blinded trial, tafenoquine was found to cause a slightly higher incidence of psychiatric symptoms than mefloquine, including abnormal dreams, agitation, anxiety, amnesia, and paranoia [238], with other reported symptoms including insomnia and depression [239].

### Other anti-malarials

In contrast to the quinoline class of anti-malarials, there is a paucity of data on the psychiatric effects of newer anti-malarial classes, including the tetracyclines, and artemisinins. What evidence does exists suggests these drugs cause a narrower range, and typically fewer and less extreme psychiatric symptoms than do the anti-malarial quinolines.

For example, in contrast to trials of tetracycline in combination with mefloquine [240], early trials of tetracycline monotherapy for treatment of malaria reported no psychiatric adverse effects [104]. Early trials of minocycline monotherapy for treatment and prophylaxis also reported no psychiatric adverse effects [241]. Indeed, reviews of the use of tetracyclines [242] and specifically doxycycline [243] in malaria do not specifically mention adverse psychiatric effects, and one recent review suggested these are “nearly nonexistent” [244]. Despite this, a number of studies comparing doxycycline to mefloquine report psychiatric symptoms such as insomnia and nightmares occurring, albeit less commonly than with mefloquine [245, 246]. Additionally, a recent case series has directed attention to the potential for rare anxiety and suicidality from doxycycline [247].

In contrast, pyrimethamine, typically used in combination with sulfadoxine, appears to have few psychiatric effects. Recent reviews do not specifically mention adverse psychiatric effects from the drug [248, 249], although early observational studies suggested the combination presented a risk of insomnia and depression, although less commonly than from quinoline anti-malarials [250]. A recent trial of sulfadoxine-pyrimethamine against mefloquine also noted sleep disturbance occurring with use of the drug, although similarly less commonly than with mefloquine [251].

Likewise, the drug combination atovaquone-proguanil, commonly marketed under the trade name Malarone^®^, also appears to have few psychiatric effects. As with the other drugs mentioned in this section, recent reviews also do not specifically mention adverse psychiatric effects from the drug [252]. Although one review suggested that atovaquone-proguanil is “the best tolerated of all antimalarial medications”, psychiatric symptoms, particularly sleep disturbance and abnormal dreams, do occur with use of the drug, although again less commonly than with quinoline anti-malarials including mefloquine [253] and chloroquine-proguanil [254]. Additionally, and unlike as is the case with mefloquine [190] and with quinacrine before it [152], such psychiatric symptoms from atovaquone-proguanil are not considered prodromal or predictive of more serious neuropsychiatric adverse effects, and do not mandate discontinuation of the drug at their onset.

Lastly, and particularly given suggestive evidence of CNS toxicity from the artemisinins [255–259], psychiatric effects from anti-malarials of this class, including the semi-synthetic derivatives artemether, artesunate, and dihydroartemisinin, should be considered plausible in spite of a paucity of observational and experimental evidence of this effect. The ascertainment of psychiatric effects from the artemisinins is made particularly challenging both by their widespread use in the treatment of symptomatic malaria [260, 261], and by their ubiquitous combination with other anti-malarial drugs, including the quinoline drugs amodiaquine and piperaquine [262], which are themselves not well studied for psychiatric effects. Although a recent systematic review found consistently higher rates of psychiatric effects, particularly sleep disturbances, with artemisinin-based combination therapies containing mefloquine than with those containing other drugs, these cited studies were not designed to assess the unique effects of artemisinins [260], and no prospective studies have assessed the psychiatric effects of artemisinin monotherapy alone.

## Conclusions

This review has demonstrated that malaria, regardless of its severity, has historically been associated in the literature with certain common psychiatric effects that resemble those now known to be caused by certain anti-malarial drugs. This review has also demonstrated that as awareness of the adverse psychiatric effects of anti-malarial drugs has grown in the modern era, the number of psychiatric effects commonly attributed in the literature to malaria, and particularly to less severe forms of the disease, has steadily decreased.

Earlier historical eras, when the psychiatric effects of malaria were considered far broader than they are today, coincided with a period of ubiquitous exposure to quinolines, both as prophylaxis and in subsequent treatment of disease. Similarly, more modern eras, in which the psychiatric effects of malaria have been generally considered to be more limited, have coincided with a period of more widespread use of alternative classes of anti-malarial drugs less commonly associated with psychiatric effects. This ecological trend suggests the distinct possibility that some psychiatric effects previously attributed to malaria may have been due in whole or in part to the effects of quinoline anti-malarials.

While malaria, and particularly in its modern definition of CM, is undoubtedly associated with a risk of cognitive impairment, the insights presented in this article should draw attention to the potentially significant confounding effects of anti-malarial exposure in the causal attribution of certain other psychiatric symptoms unique to malaria. This suggests a need to critically reevaluate the existing evidence base for the potential effects of such confounding.

The insights presented here should also underscore the importance both of designing future studies of the psychiatric effects of malaria to control for such confounding as much as is feasible, and also of more thoroughly assessing and characterizing those effects potentially attributable to anti-malarials, including those drugs currently under development and testing, and planned for use in global malaria control efforts, informed by knowledge of their common adverse psychiatric effects.

## Abbreviations

ADEM: acute disseminated encephalomyelitis
CM: cerebral malaria
CNS: central nervous system
DCA: delayed cerebellar ataxia
PMNS: post-malaria neurological syndrome
SMA: severe malarial anaemia
WWI: World War I
WWII: World War II
